# Mutation of *Elfn1* in Mice Causes Seizures and Hyperactivity

**DOI:** 10.1371/journal.pone.0080491

**Published:** 2013-11-27

**Authors:** Jackie Dolan, Kevin J. Mitchell

**Affiliations:** Smurfit Institute of Genetics and Institute of Neuroscience, Trinity College Dublin, Dublin, Ireland; University College London, United Kingdom

## Abstract

A growing number of proteins with extracellular leucine-rich repeats (eLRRs) have been implicated in directing neuronal connectivity. We previously identified a novel family of eLRR proteins in mammals: the *Elfns* are transmembrane proteins with 6 LRRs, a fibronectin type-3 domain and a long cytoplasmic tail. The recent discovery that Elfn1 protein, expressed postsynaptically, can direct the elaboration of specific electrochemical properties of synapses between particular cell types in the hippocampus strongly reinforces this hypothesis. Here, we present analyses of an *Elfn1* mutant mouse line and demonstrate a functional requirement for this gene *in vivo*. We first carried out detailed expression analysis of *Elfn1* using a β-galactosidase reporter gene in the knockout line. Elfn1 is expressed in distinct subsets of interneurons of the hippocampus and cortex, and also in discrete subsets of cells in the habenula, septum, globus pallidus, dorsal subiculum, amygdala and several other regions. Elfn1 is expressed in diverse cell types, including local GABAergic interneurons as well as long-range projecting GABAergic and glutamatergic neurons. Elfn1 protein localises to axons of excitatory neurons in the habenula, and long-range GABAergic neurons of the globus pallidus, suggesting the possibility of additional roles for Elfn1 in axons or presynaptically. While gross anatomical analyses did not reveal any obvious neuroanatomical abnormalities, behavioural analyses clearly illustrate functional effects of *Elfn1* mutation. *Elfn1* mutant mice exhibit seizures, subtle motor abnormalities, reduced thigmotaxis and hyperactivity. The hyperactivity is paradoxically reversible by treatment with the stimulant amphetamine, consistent with phenotypes observed in animals with habenular lesions. These analyses reveal a requirement for Elfn1 in brain function and are suggestive of possible relevance to the etiology and pathophysiology of epilepsy and attention-deficit hyperactivity disorder.

## Introduction

The initial connectivity of the nervous system is established through a series of processes encoded by a genetic program. Growing axons must be guided along stereotyped pathways, via intermediate targets, and must select a general target region to invade and specific cell types within it as synaptic partners [1]. The elaboration of specific types of synapses must also be controlled, in a manner that matches pre- and post-synaptic partners [2].

Leucine-rich repeat (LRR) proteins comprise an important family of molecules involved in the molecular specification of these processes [3]. Genetic evidence in flies revealed important roles for several LRR proteins in axonal pathfinding and in the selection of synaptic targets in the neuromuscular and visual systems [4], [5], [6], [7], [8], [9], [10]. Parallel discoveries in vertebrate systems revealed functions for many LRR proteins in synaptogenesis [11], [12], [13], [14], [15], [16], [17], [18], [19], [20], [21]. The importance of this class of genes for brain development is reinforced by the implication of mutations in various LRR genes in a range of neurological and psychiatric diseases (e.g., [22], [23], [24], [25], [26], [27], [28], [29], [30], [31], [32], [33]).

To get a comprehensive picture of the LRR superfamily, we previously carried out a bioinformatics survey, mining the proteomes of human, mouse, Drosophila melanogaster and C. elegans, to define the entire complement of extracellular LRR proteins in each of these organisms [34]. This screen identified 135 proteins in mammals, 66 in fly and 29 in worms. These could be classified into several groups based on the presence of additional protein motifs such as immunoglobulin (Ig) domains or intracellular Toll/interleukin 1 receptor (TIR) domains. Within these large groups, multiple subfamilies are apparent. Based on our expression screen and on previous reports, it is striking how many eLRR proteins are expressed in the developing nervous system in highly selective patterns (e.g., [35], [36], [37]), consistent with a possible role in encoding connectivity information [3], [34].

One major group of eLRR proteins includes extracellular immunoglobulin and/or fibronectin type 3 (FN3) domains, in addition to the LRRs [38]. Such domains are also found in the immunoglobulin superfamily, which itself includes many genes involved in neural development [39]. The LRR_Ig/FN3 group includes many subfamilies with known roles in neural development, including the Ntrk, Lrfn, NGL, LINGO, Lrig, Flrt and AMIGO subfamilies, among others. Both the number of distinct subfamilies and the number of overall members of this group have expanded dramatically in vertebrate evolution.

Among this group, we identified a novel family of two genes, which we named Elfn1 and 2 (for extracellular Leucine-rich repeat Fibronectin domain proteins). These proteins are characterised by a signal peptide, 6 LRR repeats, an LRR-CT and an FN3 domain extracellularly, a TM domain and a long cytoplasmic tail. The cytoplasmic tail contains several conserved motifs, one of which has recently been identified as a PP1 docking site [40]. *In situ* hybridisation revealed broad expression of Elfn2 in pre- and postnatal mouse brains, but much more restricted expression of Elfn1. Specifically, it was apparent in a subset of interneurons in the cortex and hippocampus and in a number of discrete subcortical areas, including the globus pallidus and habenula [40].

A recent study by Sylwestrak et al. found that Elfn1 is specifically expressed in somatostatin-positive interneurons in the hippocampus, particularly in the strata oriens and lacunosum moleculare (O-LM interneurons) [41]. Antibody staining revealed localisation of Elfn1 protein to the dendrites of these cells in culture, and enrichment at sites of excitatory synapses onto these cells. Knockdown and ectopic expression experiments demonstrated that Elfn1 determines the electrical properties of the synapses that pyramidal cells make onto those interneurons, compared to the synapses that the same pyramidal cells make onto parvalbumin-positive interneurons that do not express Elfn1. In particular, Elfn1 directs the elaboration of a facilitating synapse with low release probability. By directing this cell-pair-specific synapse type, Elfn1 influences the temporal dynamics of O-LM interneuron recruitment by pyramidal cell activity, which in turn is likely to exert considerable influence on activity and information flow in hippocampal microcircuits and possibly on behaviour.

To explore the possible functions of Elfn1 *in vivo*, we have analysed a knockout mouse where the *Elfn1* coding region is replaced with the gene encoding β-galactosidase (LacZ). We confirm expression of Elfn1 in specific subsets of interneurons in hippocampus and cortex and also demonstrate expression in various other cell types and brain regions, including long-range projecting GABAergic cells in globus pallidus and glutamatergic cells in habenula. In addition, we show that Elfn1 protein is present in axons *in vivo*, as well as in dendrites. While gross anatomical analyses did not reveal any obvious neuroanatomical abnormalities, behavioural analyses clearly illustrate functional effects of *Elfn1* mutation. *Elfn1* mutant mice display seizures, subtle motor abnormalities, hyperactivity and altered thigmotaxis behaviour. The hyperactivity is paradoxically reversible by treatment with the stimulant amphetamine, consistent with phenotypes observed in animals with habenular lesions and suggestive of possible relevance to attention-deficit hyperactivity disorder.

## Methods

### Ethics statement

All procedures were performed in accordance with Statutory Instrument No. 566 of 2002 (Amendment of Cruelty to Animals Act, 1876). All experiments were approved by the Animal Research Ethics Committee of Trinity College Dublin and carried out under licence B100/3533 issued by the Department of Health to KJM.

### *In situ* hybridisation

Digoxigenin-labelled antisense cRNA probes for *in situ* hybridisation of *Elfn1* were designed to encompass a section of coding sequence and 3′UTR >500bp in length. Briefly this involved TA cloning of PCR products into the TOPO vector (Invitrogen) and simultaneous synthesis and Dig-labelling of RNA transcripts from linearised vector using T7- or Sp6- RNA polymerase. Detailed information on probes is available upon request.

*In situ* hybridisation was carried out on vibratome-sectioned C57Bl6 mouse brains (Jackson Laboratories). For P0, brains were dissected out prior to immersion in 4% paraformaldehyde-PBS (PFA) at 4°C. Fixed brains were embedded in 3% agarose and 70 µm sections were obtained on a vibratome (VT1000S Leica). Sections were washed twice in PBST (PBS containing 0.1% Tween-20), permeabilised in RIPA buffer (150 mM NaCl, 50 mM Tris-HCl pH 8.0, 1 mM EDTA, 1% Nonidet-P40, 0.5% sodium deoxycholate, 0.1% SDS) and postfixed in 4% PFA and 0.2% gluteraldehyde. Hybridisation was performed in a humidified environment overnight at 65°C with 1 µg/ml labeled probe in hybridisation buffer (50% formamide, 5X SSC pH 4.5, 1% SDS, 50 µg/ml yeast tRNA, 50 µg/ml heparin). Posthybridisation washes were completed at 65°C using solution I (50% formamide, 5X SSC pH 4.5, 1% SDS) and solution III (50% formamide, 2X SSC pH 4.5, 0.1% Tween-20) and at room temperature using TBST (TBS containing 1% Tween-20). Brain sections were incubated for >1 hr in blocking buffer (TBST, 10% heat-inactivated sheep serum). Immunodetection was carried out in blocking buffer at 4°C overnight using a phosphatase conjugated anti-digoxigenin antibody (Roche) at a 1∶2000 dilution. Following antibody incubation extensive TBST washes were performed. Sections were equilibrated in NTMT (100 mM Tris-HCl pH 9.5, 100 mM NaCl, 50 mM MgCl_2_, 1% Tween-20) prior to colourimetric detection using 2 µl/ml NBT/BCIP (Roche) in NTMT. Sections were mounted on Superfrost glass slides (VWR international) and analysed with an Olympus IX51 microscope.

### Genotyping of mice

Approximately 3 mm of fresh tail tissue was digested in 200 µl Boston Buffer (50 mM Tris pH 8.0, 50 mM KCl, 2.5 mM EDTA, 0.45% NP40, 0.45% Tween20) with 14-22 mg/ml Proteinase K overnight at 56°C. 1 µl was used as template for PCR with the following primers ; Elfn1forward (5′ TGCAGCAGAGAGTACTTCAG 3′), Elfn1reverse (5′ TCTCACACACCGAGAGCTTG 3′), LacZreverse (5′ GTCTGTCCTAGCTTCCTCACTG 3′).

### Immunohistochemistry

Newborn (P0) mice were collected on day of birth and decapitated. Brains were collected and dissected in ice-cold PBS before fixation at 4°C for 4–24 hours in either 4% paraformaldehyde (PFA) or 2% PFA;0.2% glutaraldehyde. Fixed brains were embedded in agarose (Sigma), 4% w/v, in PBS. Free-floating sections (50–70 µm) were collected using a vibrating microtome (Leica VT1000S) and washed twice for 10 minutes each, in PBS containing 0.2% triton X-100. Sections were then incubated in blocking buffer (10% normal serum in PBS) for 1 hour at room temperature, with gentle agitation. Incubation in primary antibody was carried out in PBS at 4°C with gentle agitation, for 24–48 hours. After removal of primary antibody, sections were washed three times for 10 minutes each in PBS at room temperature. Sections were then incubated for 2–4 hours in the dark with a fluorescently tagged secondary antibody, diluted in PBS. This was followed by two washes in PBS of 10 minutes each, and a 10 second incubation in 4′,6-diamidino-2-phenylindole (DAPI) diluted 1∶5000 in dH_2_O. Sections were washed briefly in PBS and mounted onto slides with Aqua Polymount (Polysciences Inc.). Results were viewed with an epifluorescence microscope (Zeiss Axioplan2, Carl Zeiss Ltd. UK) or a laser scanning confocal microscope (Leica, DMRE). Primary antibodies used in this study included pan-Elfn (anti-Elfn2; Sigma HPA000781; 1∶50), anti-Ctip2 (Abcam 25B6; 1∶250), anti- neurofilament (DSHB 3H2; 1∶250), anti-somatostatin (Millipore MAB354, 1∶50), anti-Parvalbumin (Swant 235; 1∶1000), anti-β-galactosidase (MP Biomedicals 08559762; 1∶10,000) and anti-substance P (Chemicon MAB356; 1∶125). Quantitative analysis of Elfn1 expression patterns was performed on 3D confocal fluorescence image stacks using the cell counter plugin in ImageJ. Counts were obtained from three sections and the mean count calculated.

### Animal Husbandry

Mice were maintained and bred in a 12 hr light/12 hr dark cycle, in a specific pathogen free unit. Mice used for experiments was an inbred strain of C57BL/6JolaHsd (Harlan) and a strain of C57BL/6 mice with a complete deletion of the *Elfn1* coding region obtained from KnockOut Mouse Project (KOMP).

### Behavioural analyses

All behavioural tests within an experiment were carried out at the same time of day (either 9am-1pm or 2–4pm). Only male mice were tested and were handled for 3 days prior to each behaviour test. Animals were brought to the test room 15 minutes before each experiment to allow habituation to the surroundings.

### Wire-hang test

To evaluate muscle strength and co-ordination in *Elfn1^−/−^* and *Elfn1^+/−^* mice, front- and hind- paw strength was evaluated on the wire-hang test. The apparatus consisted of a horizontal steel wire (26 cm long, 0.2 cm diameter), suspended between 2 wooden poles (19.5 cm high) above a padded surface. The test consisted of 3 one-minute trials. On each trial the mice were suspended from the wire by the front 2 paws only. The mice were released and any mouse that crossed the wire to the poles at either end was deemed successful. The length of time taken to reach a pole was recorded.

### Open-field test

The first set of open field tests were conducted in 2 rectangular plastic boxes (30 cm×50 cm×18 cm) and (34 cm×60 cm×20 cm) with open tops and a grid marked out on the floor. The smaller box contained a grid consisting of 15 squares (10 cm×10 cm each) and was black in colour. The larger box contained 18 squares (9.5 cm×11 cm each) and was cream in colour. Boxes were placed on a table and against a wall in the behaviour room, away from direct fluorescent lighting. Activity measurements involved manual observation of grid line crosses with a counter. Immediately after placement of a mouse in the field, the number of line crosses were noted over 3 minutes. 16 mice were tested, 8 heterozygous and 8 homozygous mutant at 4–5 months old. Testing was carried out on consecutive days in the same box to observe habituation. Further testing in the second open-field box was carried out to evaluate novelty-induced behaviour.

A second cohort of mice were tested in a new open field area with video tracking equiptment and activity was analysed using EthoVision. The open field box was cream in colour, and placed within a white circular tank (60 cm in height) above which the video camera was positioned. This environment was more exposed and more brightly lit than in the previous experiment. Ten mice of each genotype (−/− and +/−) were tested and distance travelled and zone preference (peripheral or centre) were recorded over 10 minutes in an open field box (34 cm×53 cm×18 cm). Testing was carried out on consecutive days in the same box to observe habituation behaviours.

### Drug Administration

Amphetamine was dissolved in saline and injected intraperitoneally. Two concentrations were used, 0.5 mg/kg and 2 mg/kg. Saline solutions were administered to control anmals. Animals were injected 15 minutes before testing in the open field (as detailed above).

### Seizure observation

Mice were placed in an empty cage with clean litter and observed for seizure behaviour over a 3 minute time period [42]. Two genotypes were assessed, *Elfn1^−/−^* mice and *Elfn1^+/−^* and mice were aged 6–7 months old. Length of time in seconds was noted for each seizure from the first clonus behaviour to when the mouse regained motor control.

Seizures were described using a modification of Racine's seizure scoring system [43] by Croll et al. [44]. In this paper, Croll and colleagues attribute scores from 1–8 with increasing severity of the seizure. A score of 1 represented freezing or staring, 2 represented nodding, gnawing, facial atomatisms, or mild tremors, 3 represented unilateral clonus, 4 represented bilateal forelimb clonus, 5 represented severe seizures with prolonged loss of postural control or prolonged tonus, 6 represented status epilepticus defined as 10 minutes or more of continous or closely spaced seizures with no return to normal behaviour, 7 represented status epilepticus which included a stage 5 seizure, and 8 represented seizure-induced death.

### Data analysis

Group values are means and comparisons were performed using Student's *t*-test to determine the significance of differences between the two groups using GraphPad. Results were considered significant if *p*<0.05. Post-hoc analysis of the motor strength and coordination results was carried out using Fisher's exact test.

## Results

### Characterisation of the *Elfn1* knockout mouse line

**Figure 1:**
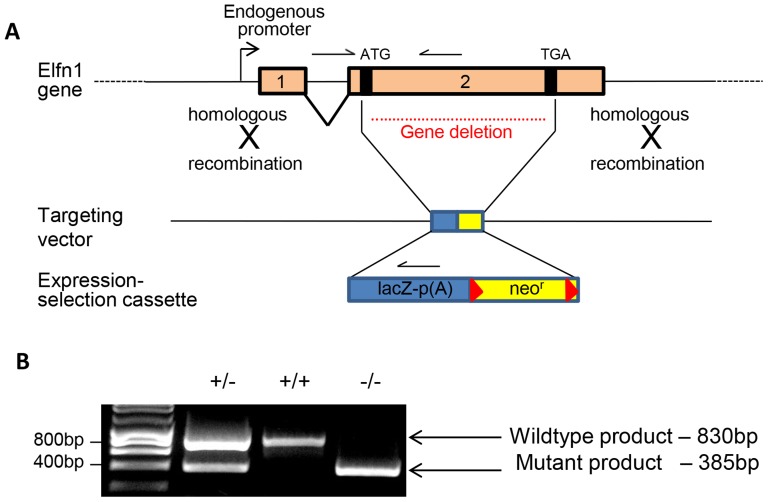
Elfn1 targeted allele. (A) Schematic of the targeting vector and the Elfn1 locus (adapted from the Knockout Mouse Project (KOMP); null allele targeting strategy (www.velocigene.com/komp/detail/10112). The full coding sequence is contained in exon 2, and this region is replaced by the expression-selection cassette. LacZ, β-galactosidase coding sequence; Neo^r^, neomycin phophotransferase; red triangles represent *loxP* sites. Genotyping primers are represented by half arrows. (B) PCR genotyping of genomic DNA from *Elfn1^+/−^*, wildtype and *Elfn1^−/−^* mice. Resultant PCR product sizes are 830bp (wildtype) and 385bp (Elfn1-targeting vector fragment).

We obtained an *Elfn1* knockout mouse line from the Knockout Mouse Project [45]. Successful deletion of *Elfn1* and replacement with the β-galactosidase (β-gal) coding sequence was confirmed, using polymerase chain reaction (PCR) of genomic DNA (Figure 1).

**Figure 2:**
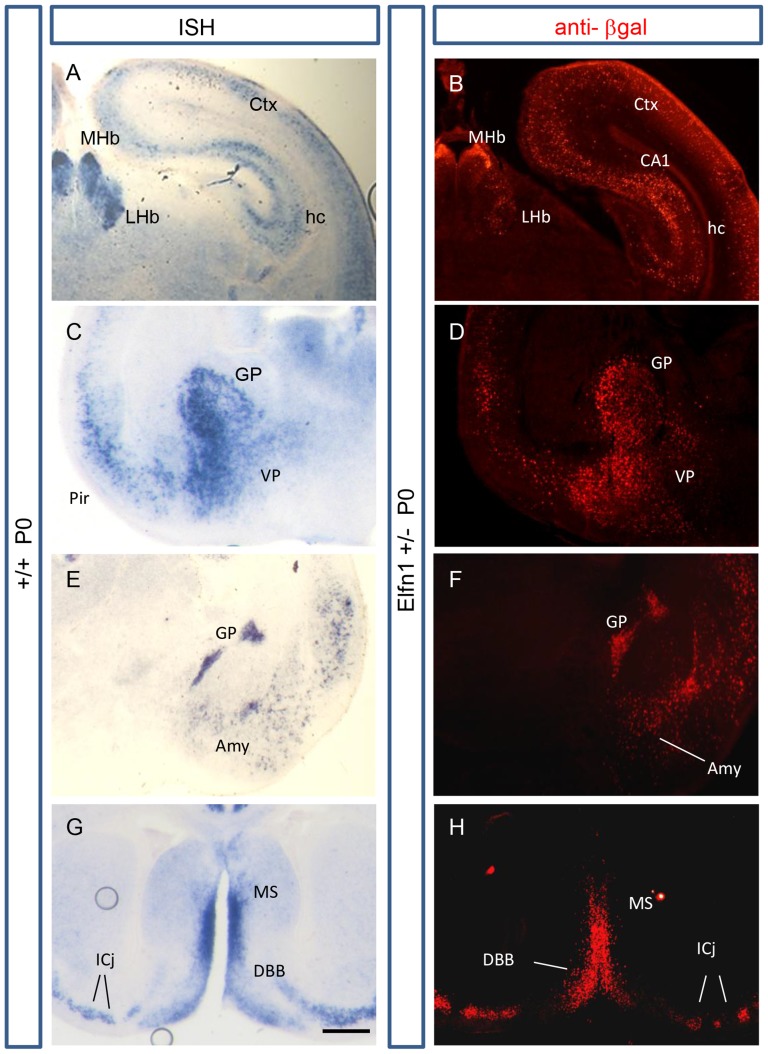
β-gal immunohistochemistry replicates *Elfn1 in-situ* hybridisation staining patterns. Comparative coronal sections of β-gal immunoreactivity in an *Elfn1^+/−^* mouse brain and *in situ* hybridisation staining using an *Elfn1* probe in a wildtype brain, both at P0. (A,B) Strong staining is evident in the dorsal MHb and the central region of the LHb. Scattered cells in the cortex and hippocampus are also observed, in a layer-specific manner in hippocampus, with particularly strong staining in the CA1 region. (C,D) Strong staining of Elfn1-positive cells are identified with both methods in the globus pallidus and ventral pallidum, with intense staining specifically in the lateral cells of the GP. (E,F) The amygdala, piriform cortex, and the caudal region of the globus pallidus exhibit comparable staining. (G,H) Intense staining in the medial septum, and to a lesser extent in the DBB, is evident. The forming Islands of Calleja also show staining with both methods of Elfn1 detection. amy, amygdala; CA1, CA1 region of the hippocampus; ctx, cortex; DBB, diagonal band of Broca; GP, globus pallidus; hc, hippocampus; ICj; islands of Calleja; LH, lateral hypothalamus; LHb, lateral habenula; MHb, medial habenula; MS, medial septum; Pir, pirform cortex; VP, ventral palidum. Scale bar: 300 microns.

The reliability of the β-gal reporter in the *Elfn1* knockout mouse was investigated by comparing the expression of β-gal in *Elfn1* heterozygous mice with *Elfn1 in situ* hybridisation results obtained from wildtype mice, both at P0. β-gal expression showed the same overall patterns as those obtained by *in situ* hybridisation, with strong expression observed in the globus pallidus, ventral pallidus, septal nuclei and in discrete nuclei within the habenula (Figure 2). Immunoreactivity was also evident in a subset of cells in the hippocampus and the cortex, as well as the diagonal band of Broca, islands of Calleja and amygdala (Figure 2). *In situ* hybridisation data from the Allen Brain Atlas showed that the regional and cell-type specificity of *Elfn1* expression is maintained in adults (Figure S1).

### *Elfn1* expression in interneurons of the hippocampus and cortex

**Figure 3:**
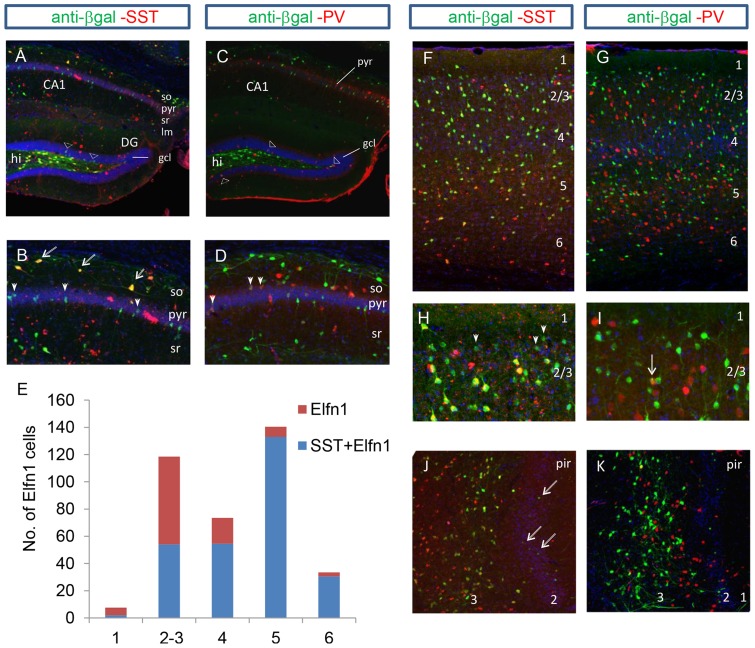
Elfn1 expression in the hippocampus and cortex. (A,B) B shows a close-up of the CA1 region in A. In the adult hippocampus, the β-gal reporter is expressed in the so, pyr, sr and lm layers and also the granule cell layer (A; open arrowheads) and hilus of the dentate gyrus. Double immunoreactivity with β-gal and SST showed co-expression in the so, sr and hilus of the dentate gyrus. Co-expression in the stratum oriens at CA1 occurred in cells with a horizontal orientation (B; arrows) and small vertically orientated cells in the pyramidal cell layer are negative for SST staining (B; closed arrowheads). (C,D) A small number of cells (0.9%) co-labelled for β-gal and PV and were found in so, pyr, sr and gcl (C; open arrowheads) of the dentate gyrus. (D) Close-up of the CA1 region shows double labelling of β-gal and PV in a number of small cells located in the pyramidal cell layer (closed arrowheads). (E) Quantification of the number of cells in each layer of the adult somatosensory cortex that label for both β-gal and SST and cells that express β-gal only. (F,G) Double immunofluorescent staining for β-gal and SST (F) and β-gal and PV (G) across the 6-layered adult somatosensory cortex. (H) Close up of layers 1 and 2/3 in F showing β-gal containing cells that do not express SST, with a smaller cell body than the surrounding double-labelled cells (closed arrowheads). (I) A small number of cells co-express β-gal and PV in the cortex (arrow). (J,K) β-gal-positive cells are most abundant in layer III of the piriform cortex, and in scattered cells in the pyramidal-rich layer II (J; arrows). 77% of β-gal-containing cells were positive for SST while only 3% contained PV. ctx, cortex; CA1, CA1 region of the hippocampus; DG, dentate gyrus; gcl, granule cell layer of the dentate gyrus; so, stratum oriens; sr, stratum radiatum; lm, lacunosum-moleculare; PV, Parvalbumin; Pir, piriform cortex; pyr, pyramidal cell layer; SST, somatostatin.

In the hippocampus at P0, *Elfn1* expression was located in the strata oriens, radiatum, lacunosum moleculare and in scattered cells in the hilus of the dentate gyrus (Figure 2). A recent study of *Elfn1* expression in the hippocampus found co-localisation with somatostatin (SST)-positive interneurons at P14, where 96% of *Elfn1* containing cells also contained SST [41]. In this study, double-labeling with antibodies for β-gal and SST at P0 similarly identified co-expression of SST in Elfn1-positive interneurons in the hippocampus (data not shown). In the adult hippocampus, expression of Elfn1 can be seen in the strata oriens, radiatum and lacunosum moleculare (Figure 3A,C). Furthermore, we identified subsets of Elfn1-expressing cells in the pyramidal cell layer of the CA1 region and the granule cell layer of the dentate gyrus (Figure 3A,B).

In the hippocampus, 64% of cells expressing Elfn1 also contained SST (n = 82/128); the majority of these cells were localised in the stratum oriens and the hilus of the dentate gyrus (Figure 3A). Cells expressing both Elfn1 and SST in the stratum oriens and hilus had a horizontal orientation (Figure 3A,B), as previously observed at P7 by Sylwestrak and Ghosh (2012). Double immunohistochemistry revealed that 11% of Elfn1-expressing cells also contained parvalbumin (PV); these were localised in the stratum oriens, pyramidal cell layer, stratum radiatum and the granule cell layer of the dentate gyrus (Figure 3C,D). Most of these cells displayed a vertical orientation with a comparatively small cell body size (Figure 3B,D).

In the neocortex and piriform cortex at P0, Elfn1-expressing cells were distributed unevenly across layers, with highest concentration in layers 4 and 5, and less expression in layers 2 and 3, while only a small number of cells were observed in layers 1 and 6 (Figure S2). In the 3-layer piriform cortex, Elfn1-expressing cells appeared mainly resticted to layer 3 and the endopiriform cortex. A small number of positive cells could also be seen in layer 1, and the pyramidal cell-rich layer 2 (Figure S2).

Quantitative analysis of Elfn1 expression in the adult cortex revealed that the highest number of Elfn1-containing cells localise to layer 5, layers 2–3 and layer 4. Only a small number of cells in layers 1 and 6 express Elfn1 (Figure 3E). Co-localisation analyses revealed that 77% of Elfn1-expressing cells also contained SST (n = 287/373), but the proportion of SST co-expressing cells varied by layer. In particular, layer 5 contained the highest proportion of Elfn1 and SST co-expressing cells (Figure 3E). Only 0.9% of Elfn1-expressing cells in cortex contained PV (Figure 3G,I). The remaining 22.1% of Elfn1-expressing cells express neither SST nor PV and were highly concentrated in layers 2–3. Interestingly, the cell bodies of these cells appear smaller than the surrounding Elfn1 and SST co-expressing interneurons (Figure 3H).

In the piriform cortex, 77% of Elfn1-positive cells contained SST and 3% contained PV (Figure 3J,K). While the majority of cells expressing Elfn1 were mainly resticted to layer 3, a small subset of Elfn1-expressing cells localised to the pyramidal cell-rich layer 2. The cell bodies of these cells appear smaller in size than the surrounding SST- and Elfn1-containing interneurons (Figure 3J).

### Elfn1 expression in subcortical structures

#### Habenula

**Figure 4:**
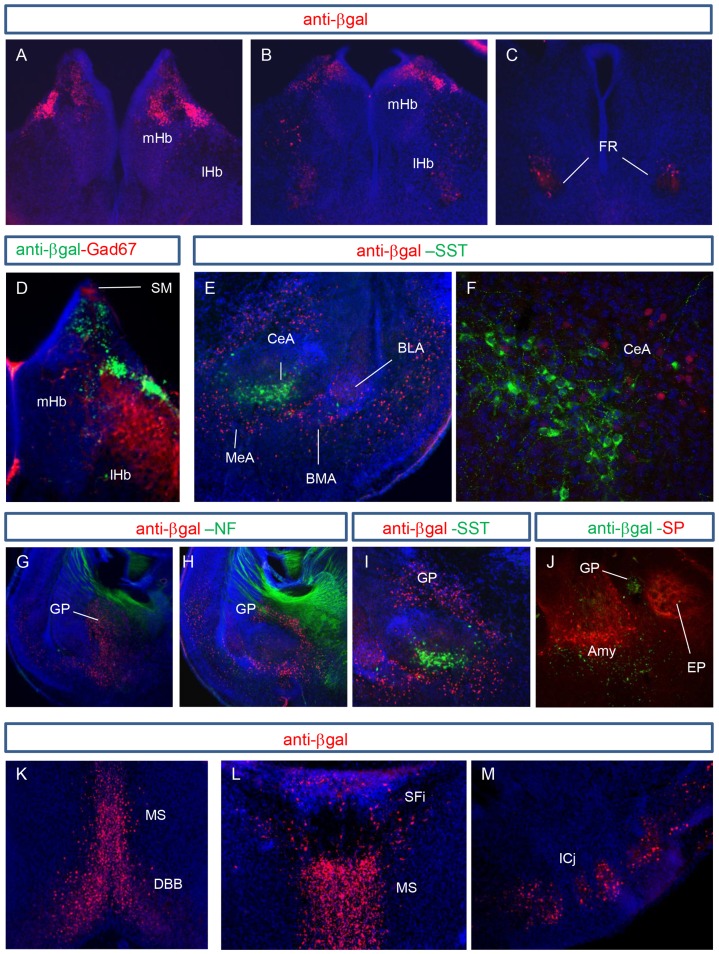
Elfn1 expression in subcortical structures of the mouse brain. (A–C) The β-gal reporter is expressed throughout the rostrocaudal extent of the habenula. In a rostral coronal section (A) staining is limited to the dorsolateral MHb and absent from the LHb. Further caudally staining is restricted to a smaller subset of cells in the dorsolateral MHb, and in scattered cells of the central LHb (B). In a caudal-most section, β-gal staining is absent from the MHb, and a few positive cells are located in the ventral LHb at the fasciculus retroflexus (C). (D) GAD67 staining is localised to the stria medullaris, and the lateral habenula. There is no overlap in β-gal and GAD67 staining in the MHb. (E) β-gal expression is evident in the medial (MeA), basomedial (BMA) and basolateral amygdala (BLA). Scattered cells in the central (CeA) are also positive for β-gal. Double immunoreactivity with β-gal and SST antibodies identify that expression of these two proteins are largely in separate regions of the amygdala. (F) A magnified confocal image of (E) shows that βgal-positive cells do not contain SST. (G,H) β-gal is expressed throughout the rostrocaudal extent of the globus pallidus. In a rostral section of the GP there is a strong lateral to medial gradient (G) and staining is also evident thoughout the more elongated structure in caudal sections (H). (I) Cells positive for β-gal do not colocalise with cells expressing SST in the globus pallidus. (J) β-gal staining is not observed in the entopeduncular nucleus which is highly immunopositive for Substance P (SP). (K–M) β-gal is also expressed in the medial septum (MS), diaganol band of Broca (DBB), septofimbrial nucleus (SFi) and in the islands of Calleja (ICj). Amy, amygdala; EP, entopeduncular nuleus; FR, fasciculus retroflexus; GP, globus pallidus; lHb, lateral habenula; mHb, medial habenula.

Immunolabelling for β-gal was evident throughout the rostrocaudal extent of the habenula, where it was restricted to the dorsal part of the medial habenula and in scattered cells of the lateral habenula (Figure 4A–C). In the rostral portion of this structure, labelled cells were found in the dorsolateral medial habenula, and were absent from the LHb (Figure 4A). More caudally, expression of β-gal was further restricted to a discrete subset of cells in the dorsal-most region of the MHb, while a small number of scattered positive cells became evident in the central part of the LHb (Figure 4B). Further caudally, there were no labelled cells in the MHb and expression appeared to be localised only to the ventral region of the LHb, surrounding the fasciculus retroflexus (Figure 4C).

It had previously been noted that there are no GABAergic cells in the MHb [46] and in this study, double staining for GAD67 and β-gal showed no cellular overlap (Figure 4D). Therefore, in the MHb Elfn1 is not expressed in interneurons.

#### Amygdala

β-gal immunoreactivity was detected in cells throughout the amygdala, especially in the medial (MeA) and basolateral (BLA) nuclei and in a small number of cells in the central amygdaloid nucleus (Figure 4E, F). SST-containing cells have been identified in the central amygdala (CeA) [47] as well as the basolateral (BLA) nucleus [48]. SST staining was strong in the CeA and in much fewer cells of the BLA, but did not overlap immunoreactivity with β-gal (Figure 4F).

#### Globus pallidus

The globus pallidus (GP) exhibited very high expression of Elfn1, during development at E15 [34] and at P0 (Figure 4G–I). β-gal-positive cells could be seen throughout the the rostro-caudal extent of this structure (Figure 4G,H). In rostral sections, the lateral-most part displayed the strongest signal of β-gal, (Figure 4G). This staining appeared to coincide with “outer” globus pallidus neurons, many of which project to striatum [49]. Double staining for SST and β-gal established that in the globus pallidus, unlike in the cortex and hippocampus, Elfn1 was not expressed in SST-positive interneurons (Figure 4I). The entopeduncular nucleus contains intense substance P staining, but cells in this region did not express Elfn1 (figure 4J). A small corridor of cells bordering this region displayed weak β-gal staining, likely to be the caudal-most part of the globus pallidus.

#### Septum and ventral forebrain structures

In the medial septal nucleus, there was a high concentration of cells positive for β-gal. The expression continued ventrally into the diagonal band of Broca to include both the vertical and horizontal limbs (Figure 4K). Scattered cells of the septofimbrial nucleus were β-gal-positive but the lateral septum and the triangular septal nucleus appeared unstained (Figure 4L). Elfn1 was also expressed in the newly-forming islands of Calleja at P2, but not in the large Insula Magna (Figure 4M). These structures are composed mainly of GABAergic cells and are innervated by dopamine neurons of the mesencephalon and interconnected with olfactory and non-olfactory components of the basal forebrain [50].

### ELFN1 localisation to axons of the fasciculus retroflexus, the lateral IPN and the substantia nigra pars compacta

We carried out immunohistochemistry using a pan-ELFN antibody that recognises both ELFN1 and ELFN2, but cannot differentiate between the two (Sigma HPA000781). Unfortunately, widespread expression of ELFN2 in the mouse brain prohibited the direct identification of ELFN1 protein localisation. However, the *Elfn1* knockout mouse allowed for the comparison of pan-ELFN immunoreactivity in *Elfn1^−/−^* and *Elfn1^+/−^* mice. Loss of staining in the *Elfn1^−/−^* mouse identified regions of ELFN1 protein localisation.

**Figure 5:**
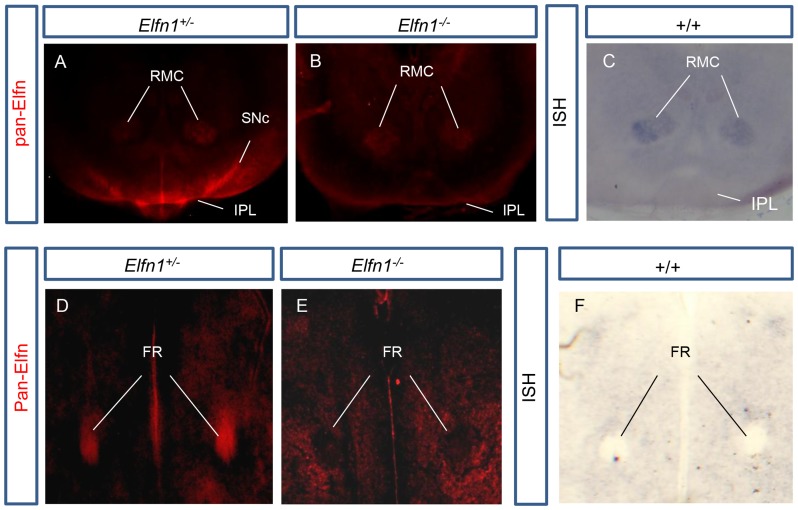
ELFN1 localises to axons of the lateral IPN, the substantia nigra pars compacta and the fasciculus retroflexus. Pan-ELFN positive staining is visible in the IPL and SNc of the *Elfn1^+/−^* (A) but not in *Elfn1^−/−^* brain (B). *In situ* hybridisation with an Elfn1 probe in this region shows that Elfn1 is not expressed by cells in the SNc or the IPL at P0 (C). (D) In the *Elfn1^+/−^* brain at P0, the pan-ELFN antibody displays strong axonal-like staining in the core of the fasciculus retroflexus (FR). (E) pan-ELFN antibody staining is absent from the FR in the *Elfn1^−/−^* brain. (F) Elfn1 *in situ* hybridisation shows that Elfn1 is not expressed by cells in the FR. FR, fasciculus retroflexus; IPL, lateral subnucleus of the interpeducular nucleus; RMC, Red nucleus magnocellular part; SNc, substantia nigra pars compacta.

*Elfn1^+/−^* coronal brain sections exhibited strong pan-ELFN immunoreactivity in the fasciculus retroflexus, the lateral IPN and the substantia nigra pars compacta that was absent in *Elfn1^−/−^* mice (Figure 5). There was no Elfn1 RNA *in situ* hybridisation staining in any of these regions at P0 and therefore we conclude that ELFN1 protein is localised to axons of the fasciculus retroflexus, the lateral subnucleus of the IPN and in axons innervating the SNc.

### Number and distribution of Elfn1-positive cells in *Elfn1^−/−^* mice

**Figure 6:**
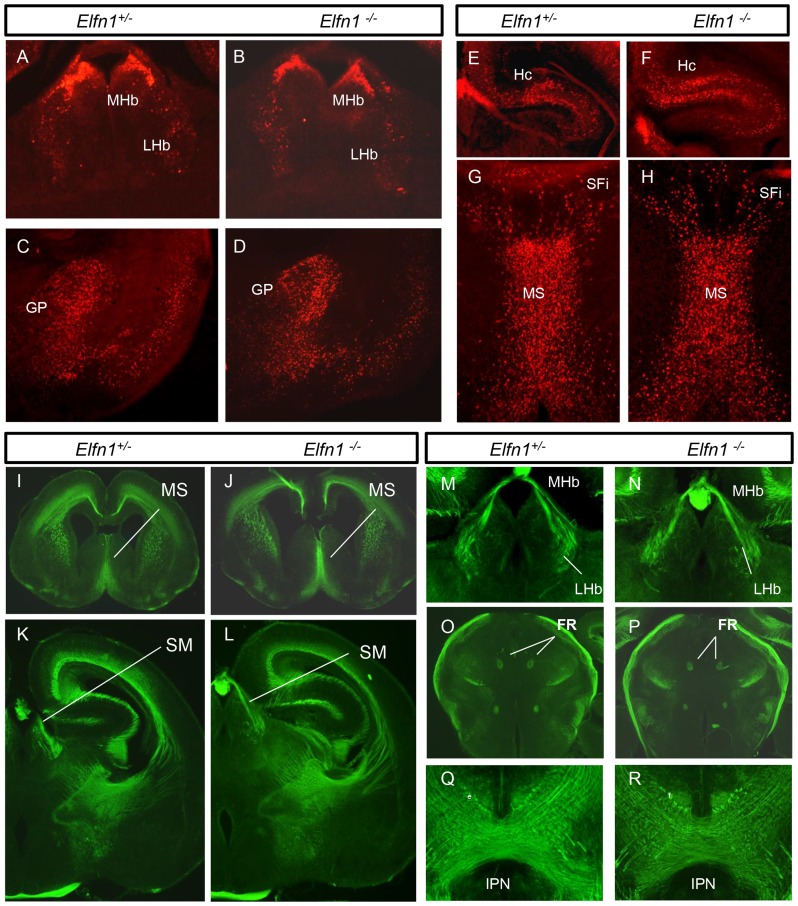
Phenotypic analysis of the *Elfn1^−/−^* mouse brain at P0. β-gal staining patterns in coronal sections of the *Elfn1^+/−^* (A,C,E,G) and *Elfn1^−/−^* (B,D,F,H) show comparable patterns indicating no obvious morphological phenotype in the *Elfn1* mutant brain (n = 4). (I–R) Neurofilament staining patterns in *Elfn1^+/−^* (I,K,M,N,Q) and *Elfn1^−/−^* (J,LN,P,R) show no apparent abnormality in connectivity (n = 4). FR, fasciculus retroflexus; GP, globus pallidus; Hc, hippocampus; IPN, interpeduncular nucleus; LHb, lateral habenula; MHb, medial habenula; MS, medial septum; SFi, septofimbrial nucleus; SM, stria medullaris; VP, ventral pallidum.

The β-gal staining patterns in *Elfn1^+/−^* and *Elfn1^−/−^* mice were compared in coronal sections of mouse brain at P0, and no gross morphological differences were detected. In all regions of Elfn1 expression, the pattern and abundance of immunoreactive cells appeared normal in the homozygous mutant (Figure 6; n = 4 of each genotype)

### Gross connectivity in *Elfn1^−/−^* mice

Neuronal processes were immunostained with a neurofilament antibody and comparable sections in *Elfn1^+/−^* and *Elfn1^−/−^* mice were analysed. No gross connectivity abnormality was identified (Figure 6; n = 4 of each genotype). Particular focus on the stria medullaris and fasciculus retroflexus revealed normal innervation patterns in the habenula and the interpeduncular nucleus (Figure 6M-R).

### Seizures in *Elfn1^−/−^* mice

Twelve *Elfn1^−/−^* animals have been witnessed having seizures during routine cage changes. These animals have been aged between 4 and 9 months old. Seizures have ranged from a score of 2 to a score 5 of the modified Racine seizure scoring system [43], [44] with one animal losing postural control during the seizure, and another displaying rapid running and jumping. Two more mice from the behavioural study (see below) exhibited a seizure (both 3 months old, score of 4 on the Racine scoring system). However, both these mice had received amphetamine, complicating interpretation.

In a three-minute observation test [42] two out of eight *Elfn1^−/−^* mice and zero out of eight *Elfn^+/−^* mice displayed seizures when placed in a clean and empty cage. All mice were 6–7 months old. One seizure represented a score of 4 in the modified Racine seizure scoring system with freezing immediately upon placement in the new cage, followed by bilateral forelimb clonus that lasted 33 seconds before motor control was regained. The second observed seizure also began with motor arrest, followed by head-bobbing and mild shaking for 22 seconds. This seizure corresponds to a score of 2 in the modified Racine seizure scoring system.

### Reduced motor coordination and strength

**Figure 7:**
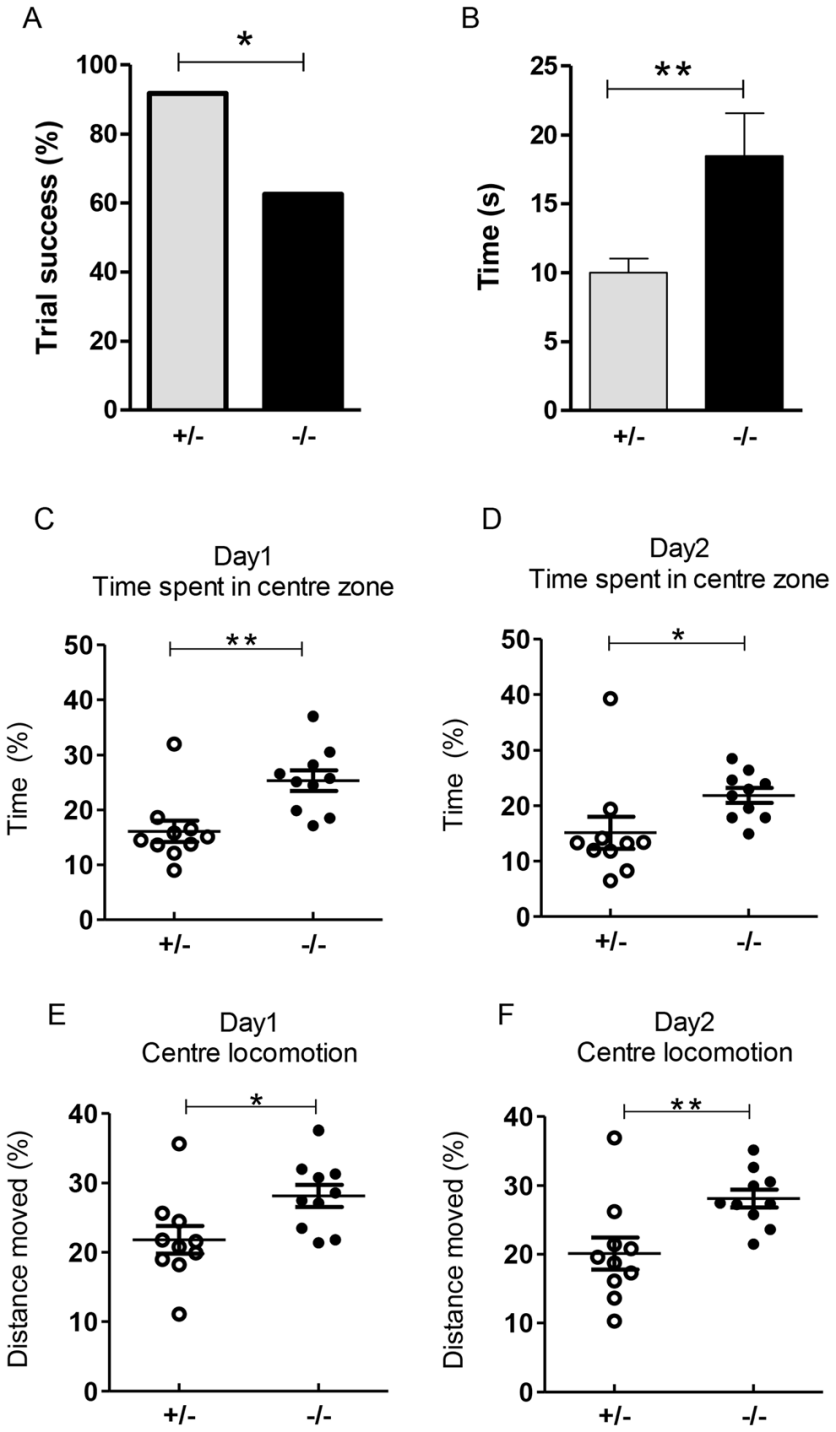
Reduced motor performance and thigmotaxis behaviour in *Elfn1^−/−^* mice. (A) *Elfn1^+/−^* mice completed a wire hang trial with a success rate of 91.67% and *Elfn1^−/−^* mice were only successful in 62.5% of the trials (n = 8/genotype; 3 trials per mouse; *p* = 0.0363). (B) The time taken to complete a successful trial was analysed and *Elfn1^−/−^* mice took considerably longer to reach a pole (*p* = 0.0054). Data expressed as mean±SEM (n = 22 for *Elfn1^+/−^* and n = 15 for *Elfn1^−/−^ mice*). Student's t test was used to compare the means. (C,D) Percentage of total time spent in the centre zone on day 1 (C) and day 2 (D). *Elfn1^−/−^* mice spent more time in the centre zone than *Elfn1^+/−^* mice on both days (*p* = 0.0031 for day 1, and *p* = 0.0496 for day 2; n = 10/genotype). (E,F) Percentage of total distance travelled in the centre zone on day 1 (E) and day 2 (F). *Elfn1^−/−^* mice travelled further than *Elfn1^+/−^* mice in the centre zone on both days (*p* = 0.023 for day 1 and *p* = 0.0076 for day 2). Student's t test used to identify statistical difference between the groups.

In the wire-hang test, *Elfn1^−/−^* mice were less successful than the *Elfn1^+/−^* mice at completing the task (62.5% vs 91.67%, n = 8 mice/genotype; 3 trials per animal; *p* = 0.0363) (Figure 7A). Moreover, when successful trials were compared across the 2 groups of mice, the time taken to reach a pole was significantly longer for the *Elfn1^−/−^* mice (18.47 s±3.13, n = 15) than the *Elfn1^+/−^* mice (10.0 s±1.02, n = 22; *p* = 0.0054) (Figure 7B).

Post hoc analysis revealed that all successful trials in heterozygous animals were completed with the use of 4 limbs. However, in 4 of the 15 successful trials carried out by *Elfn1^−/−^* mice, only the forelimbs were used to reach the poles after initial attempts to raise the hindlimbs onto the wire failed. In the 9 failed trials carried out by *Elfn1^−/−^* mice, the animals did not manage to raise their hindlimbs onto the wire, and subsequently fell off. These findings collectively indicate that *Elfn1^−/−^* mice exhibit reduced motor strength and/or coordination.

### Reduced thigmotaxis in *Elfn1^−/−^* mice

During open field behavioural testing, Ethovision analysis revealed that *Elfn1^−/−^* mice exhibited a reduction in wall-hugging behaviour or thigmotaxis. The percentage of total time spent in the centre of the open field was significantly higher in *Elfn1^−/−^* mice (25.34±1.88, n = 10) compared with the *Elfn1^+/−^* mice (16.12±1.95, n = 10; *p* = 0.0031) (Figure 7C). The percentage of total distance travelled in centre of the open field was also significantly higher for *Elfn1^−/−^* mice (28.15±1.60, n = 10) than for *Elfn1^+/−^* mice (21.82±1.99, n = 10) (*p* = 0.023; Figure 7E). This behavioural phenotype was observed again on day 2 of testing with *Elfn1^−/−^* spending more time in the centre zone (21.86±1.35 vs 15.13±2.90 n = 10/genotype; *p* = 0.0496) (Figure 7D). Locomotion in the centre zone also remained significantly higher in *Elfn1^−/−^* mice (28.12±1.30 vs 20.11±2.33, n = 10/genotype; *p* = 0.0076) (Figure 7F).

### *Elfn1^−/−^* mice exhibited hyperlocomotion

**Figure 8:**
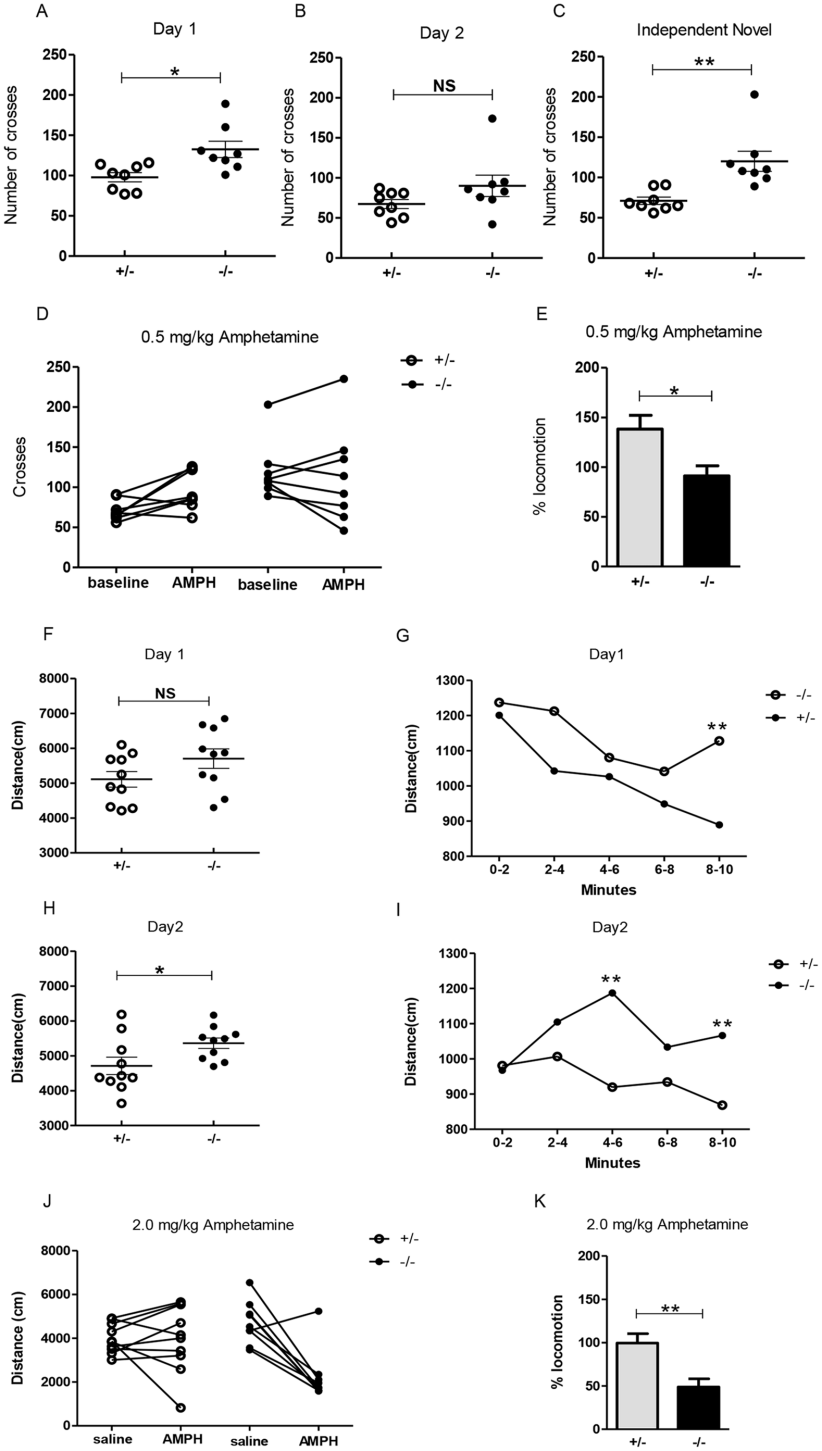
Hyperlocomotion of *Elfn1^−/−^* mice. (A–E) **Experiment 1.** (A) The number of crosses on day 1 of open field testing was significantly higher for *Elfn1^−/−^* mice than in *Elfn1^+/−^* mice (*p* = 0.01; n = 8/genotype). (B) On day 2, in the same open field, there was no significant difference between the groups (*p* = 0.138) but the mean number of crosses remained higher for *Elfn1^−/−^* mice (90.13±13.32) versus *Elfn1^+/−^* mice (67.38±5.62; n = 8/genotype). (C) Introduction to a novel environment again confirmed a higher number of crosses in *Elfn1^−/−^* mice versus *Elfn1^+/−^* mice (*p* = 0.0025; n = 8/genotype). (D) Graph shows the number of crosses before and after 0.5 mg/kg AMPH for each mouse in experiment 1 (n = 8/genotype). *Elfn1^+/−^* (white circles), *Elfn1^−/−^* (black circles). (E) The mean percentage distance travelled by *Elfn1^+/−^* and *Elfn1^−/−^* mice after 0.5 mg/kg. Student's *t* test *p* value = 0.0159. (F–K) **Experiment 2.** (F) On day 1, statistical analysis revealed no significant difference between the groups however, the mean distance travelled by *Elfn1^−/−^* mice was greater than by *Elfn1^+/−^* mice (5701±278.8 for *Elfn1^−/−^* and 5108±222.4 for *Elfn1^+/−^*, n = 10/genotype; *p* = 0.1141). (H) On day 2, in the same open field, *Elfn1^−/−^* mice travelled a significantly greater distance than *Elfn1^+/−^* mice (p = 0.0381; n = 10/genotype). (G) On day 1, the mean distance travelled by *Elfn1^−/−^* mice remained higher than for *Elfn1^+/−^* mice over the 10 minutes, and a significant difference between the genotypes is evident in the final 2 minute bin (p = 0.0021; n = 10/genotype). (I) On day 2, a significant difference between groups in the mean distance travelled is identified at the 4–6 minute and 8–10 minute time bins (*p* = 0.0024 and *p* = 0.0067 respectively). All data expressed as means, and Student's *t* test used to analyse statistical difference betwen the groups. Post-hoc analysis is not corrected for multiple testing. (J) Graph depicts differences in distance travelled by each mouse when administered saline and 2.0 mg/kg AMPH in experiment 2 (n = 10 *Elfn1^+/−^*, n = 9 *Elfn1^−/−^*). (K) The mean percentage distance travelled by *Elfn1^+/−^* and *Elfn1^−/−^* mice after 2.0 mg/kg of AMPH. Student's *t* test *p* value = 0.0029 for 2.0 mg/kg.

Activity behaviour was examined in two cohorts of male mice. The first cohort contained eight mice of each genotype (homozyous mutant and heterozygous), aged 3–4 months and the second contained ten mice of each genotype, aged 2–3 months. For the first trial of open field testing (Experiment 1), mice from cohort number 1 were placed in a rectangular box with a grid marked out on the floor. The number of line crosses was noted over 3 minutes, and taken as an indication of activity. Student's t test was used to determine the significance of differences between the two groups, and demonstrated increased locomotion in *Elfn1^−/−^* mice (132.5±10.11, n = 8) compared with the *Elfn1^+/−^* mice (97.88±5.75, n = 8; *p* = 0.01) (Figure 8A). The following day, locomotor activity was measured again in the same field to analyse activity after habituation. The Student's t test analysis showed that while activity levels decreased across both genotypes, the mean level of activity remained higher in *Elfn1^−/−^* mice (90.13±13.32, n = 8) compared with the *Elfn1^+/−^* mice (67.38±5.62, n = 8; Figure 8B) but did not reach statistical difference (*p* = 0.138). Analysis of activity in a new open field environment confirmed significantly higher activity in *Elfn1^−/−^* mice (120.1±12.55 vs 71.13± 4.53, n = 8; *p* = 0.0025) (Figure 8C).

When the activity trials from the three experiments were accumulated, providing 24 activity values for each genotype, a Student's *t* test for genotype differences confirmed that *Elfn1^−/−^* mice have substantially higher activity levels (114.3±7.62 vs 78.79±4.08, n = 24; *p* = 0.0002).

The second cohort of mice were tested for differences in activity using video tracking over an open field box and analysis with EthoVision software (Experiment 2). On day 1, evaluation of the distance travelled over a 10 minute duration using student's t test did not show statistical difference (5701±278.8 for *Elfn1^−/−^* and 5108±222.4 for *Elfn1^+/−^*, n = 10/genotype; *p* = 0.1141) (Figure 8F). However, as noted with cohort number 1, the mean activity levels of *Elfn1^−/−^* mice were higher than for *Elfn1^+/−^* mice. On the second day of testing, there was a significant increase in distance travelled by *Elfn1^−/−^* mice (5360±149.4, n = 10) when compared with *Elfn1^+/−^* mice (4710±248.6, n = 10; *p* = 0.0381) (Figure 8H).

Post-hoc analysis of day 1 revealed that when the distance was binned into 2 minute intervals, a significant difference is evident over the final 2 minutes when activity levels in *Elfn1^+/−^* mice have decreased considerably (1128.24±52.69 for *Elfn1^−/−^* and 889.42±40.36, n = 10; *p* = 0.0021) (Figure 8G). Likewise on day 2, post-hoc time-intervel analysis revealed a significant difference at 4–6 minutes (*p* = 0.0024) and again over the final 2 minutes (*p* = 0.0067) (Figure 8I). This replication experiment confirms a general but variable baseline hyperactivity in *Elfn1* mutants, with overlap in the distribution with control mice.

### Attenuation of hyperlocomotion with amphetamine

Amphetamine (AMPH) has been shown to increase extracellular dopamine levels [51]. The resultant increase in dopamine levels induces hyperactivity in normal subjects [52] and exacerbates psychotic-like hyperactivity [53], [54], [55]. Conversely, stimulant medication attenuates hyperactivity in children with attention-deficit/hyperactivity disorder (ADHD) [56], [57]. Similar effects have been observed in various animal models of ADHD [58], [59], [60], including animals with lesions in the habenula [61], [62].

We therefore examined the effects of AMPH treatments in the *Elfn1* knockout mice, starting with a low dose of 0.5 mg/kg on all mice in cohort 1 [61]. The number of crosses for each mouse one week before and 15 minutes after AMPH were compared. The percentage increase or decrease in the number of line crosses per animal after 0.5 mg/kg AMPH was evaluated in a Student's t test for genotype differences and results revealed a paradoxical attenuation of hyperactivity in *Elfn1^−/−^* mice with a mean decrease in locomotion (91.13%±10.17 n = 8) and a mean increase in locomotion, as expected, in *Elfn1^+/−^* mice (138.3%±13.87 n = 8; *p* = 0.0159) (Figure 8D,E).

The effect of a higher dose of AMPH (2.0 mg/kg) was tested with the second cohort of mice, with a different experimental design. All animals received both a saline injection (control) and an injection of AMPH (1 week apart) and were tested in the open field 15 minutes after administraton. On the first day, 5 *Elfn1^−/−^* and 5 *Elfn1^+/−^* mice received AMPH, while the other 5 *Elfn1^−/−^* and 5 *Elfn1^+/−^* mice received saline. The following week, administrations of saline and AMPH were swapped. The higher dose of AMPH produced a significant decrease in average locomotion in the *Elfn1^−/−^* mice (48.83±9.363, n = 9; *p* = 0.0029); however, the expected increase in activity of *Elfn1^+/−^* mice did not occur (99.46±10.97, n = 10) (Figure 8J,K).

## Discussion

We selected Elfn1 for functional study based on its membership of the eLRR superfamily, interesting structure and compelling expression pattern in preliminary analyses [34]. These suggested that Elfn1 might play an important role in the development of the circuitry of particular cell types and brain regions. The discovery that Elfn1 protein can direct the elaboration of specific electrochemical properties of synapses between particular cell types in the hippocampus [41] strongly reinforces this hypothesis. Our analyses of *Elfn1* mutant mice reveal a functional requirement for this gene *in vivo*, highlighted by a number of neurological and behavioural phenotypes that arise when the gene is mutated.

We previously briefly reported the distribution of Elfn1 across the brain, with expression evident in a subset of interneurons in hippocampus and cortex and also notable in discrete subregions of the globus pallidus and habenula [34]. Sylwestrak et al showed that Elfn1 is expressed most prominently in a subset of SST-positive interneurons in hippocampus at P14, with the protein localised to dendrites of these cells in culture [41]. In adults, this picture appears more complex. While the majority of Elfn1-expressing cells in stratum oriens and hilus were SST-positive, Elfn1 was also expressed in sparsely distributed cells in the pyramidal cell layer and dentate gyrus granule cell layer, many of which expressed PV and not SST.

In the cortex, Elfn1 was clearly expressed in multiple distinct cell types, both across and within layers. The largest number of Elfn1-positive cells was found in layer 5 and the majority of these were SST-positive. The proportion that was SST-positive was lower in other layers, however. Elfn1 expression was notably absent from most SST-positive cells in layer 6. The relationship between Elfn1 and SST expression is thus not exlcusive in either direction. Only very few scattered cells showed co-expression of Elfn1 and PV and 22% were negative for both SST and PV; these were notably concentrated in layers 2 and 3. The overlapping expression patterns of Elfn1 and these calcium-binding protein markers in different layers thus define new subsets of interneurons whose functions might be probed by intersectional transgene expression strategies.

Expression of Elfn1 is not restricted to locally ramifying GABAergic interneurons, however. Elfn1 is also expressed in long-range GABAergic projection neurons (e.g., in globus pallidus), and in glutamatergic projection neurons (e.g., in habenula). In addition, our antibody stainings in wild-type and homozygous mutant mice show clear ELFN1 protein localisation in axons of the fasciculus retroflexus. These findings suggest the possibility of additional axonal or presynaptic functions for the ELFN1 protein in diverse cell types. The highly specific expression pattern of Elfn1 is also maintained in adults (Figure S1), consistent with a hypothesis of ongoing as well as developmental functions for this protein.

Our anatomical analyses did not reveal any gross abnormalities in *Elfn1* homozygous mutants. The number and position of Elfn1-positive cells was not appreciably altered in any of the brain regions of prominent expression. In addition, stainings for neurofilament and other axonal markers (not shown) showed no gross differences in long-range axonal projections. If Elfn1 plays a role in directing neuronal migrations or guiding growing axons, it is not apparent from this loss-of-function analysis at this level. We cannot, however, rule out more subtle abnormalities at a finer level, including local projections within brain regions.

Despite grossly normal neuroanatomy, a functional requirement for Elfn1 is clearly revealed by the neurological and behavioural consequences of mutation of this gene. *Elfn1* homozygous mutants are prone to seizures, which have been observed in numerous animals during cage transfers, and also during a defined observation period. The seizures range in severity and manner of expression and are most probably analogous to clinical partial seizures. With the exception of homozygous mutants in an *Adam23* knockout mouse line [63], [64], which interacts with known epilepsy gene *LGI1* [23], [65], spontaneous seizures have never been observed in any of our mice in the facility over the last ten years (>30,000 mice, compared to 176 *Elfn1* homozygous mutants) or in over 60 lines assayed in a prior research project [63]. At present, we do not know the penetrance of seizures in *Elfn1^−/−^* mice; it is possible that only a proportion of these animals develop epilepsy, but equally possible that all of the *Elfn1^−/−^* mice are indeed having seizures, just not while being observed. The seizures we did observe have all been in mice greater than 4 months old, but full-lifetime observation would be required to determine whether seizures emerge with age consistently in *Elfn1* mutants.

Our analyses do not identify the neural origins of these seizures. They may relate to the function of Elfn1 in specifying the electrochemical properties of glutamatergic synapses onto interneurons in the hippocampus. SST-positive O-LM interneurons target distal dendrites of pyramidal cells and are engaged most effectively by repetitive pyramidal cell activity, due to the fact that excitatory inputs to these cells are normally strongly facilitating. RNAi-mediated knockdown of Elfn1 converted these inputs into high-release-probability, non-facilitating synapses, which are likely to generate strong but short-lived inhibition, due to synaptic depression [41]. This change may involve the presynaptic GluR6-containing kainate receptor and was found to influence the temporal dynamics of O-LM recruitment in response to pyramidal cell activity.

Whether such a change is occurring in the *Elfn1* mutants and whether it could account for the emergence of seizures in these animals are open questions. It is difficult, in the first instance, to predict the acute effects on global network activity of altering the response characteristics at this one type of synapse. It is even more challenging to predict the longer-term, emergent consequences of altering the plasticity rules at this synapse type, which may in turn affect plasticity rules more generally in the network (e.g., [66]). The lack of observed seizure activity in young mice would support the hypothesis that mutation of *Elfn1* may set into motion long-term epileptogenic processes that eventually lead to a seizure-prone brain [67]. Finally, Elfn1 is also expressed in subsets of interneurons in the hilus as well as in the neocortex and piriform cortex, and strongly in the dorsal subiculum, regions also known to exhibit epileptogenic activity [68], [69], [70].

*Elfn1* mutants show additional behavioural phenotypes that may relate to functions in other parts of the brain. The hyperlocomotion is particularly interesting, as it shows an unexpected reduction in response to amphetamine. Importantly, we have replicated this effect in two separate cohorts of mice, using two experimental set-ups. This paradoxical response to stimulants, including amphetamine, is a defining characteristic of patients with attention-deficit hyperactivity disorder (ADHD) [56], [57] and of animals thought to model aspects of the condition [58], [59], [60]. By contrast, a variety of animal models that are thought to relate more to psychosis (e.g., [71], [72], [73], [74], [75], [76]) show a form of hyperactivity that is exacerbated by amphetamine (which also exacerbates psychotic symptoms in human patients) [77]. Interestingly, mice with selective, nicotine-induced neonatal lesions in the medial habenula, a site of very strong Elfn1 expression, show a similar effect, with hyperlocomotion that is improved by amphetamine, along with impulsivity and attention deficit [61]. A similar ADHD-like phenotypic spectrum [58], [59], [60] has been observed in other mice with habenula lesions [62]. Given the tendency for these phenotypes to cluster, it will be interesting to determine whether *Elfn1* mutant mice also show impulsivity and attention deficits.

The habenula integrates signals from limbic forebrain and basal ganglia to regulate midbrain areas involved in the release of dopamine (ventral tegmental area and substantia nigra) and serotonin (median and dorsal raphe nuclei)

[78], [79]. Both the medial and lateral habenula have been shown to exhibit an indirect inhibitory influence on dopaminergic and serotonergic nuclei [80], [81], through projections to the interpeduncular nucleus and rostromedial tegmental nucleus, respectively, as well as to local GABAergic neurons in the ventral tegmental area. Projections are also made from MHb to LHb, but not in the other direction [82].

The MHb and LHb each comprise multiple subnuclei [46], [83], which receive inputs from distinct limbic and basal ganglia regions [84]. Within the medial habenula, Elfn1 expression is restricted to cells of the dorsal part, with a highly similar pattern of expression as substance P (revealed by *in situ* hybridisation) [85]. Descending projections from the MHb and the LHb show topographically organised innervation of their targets [86], which further supports the idea that distinct microcircuits produce parallel pathways of information flow to the midbrain monoaminergic nuclei. Given these complexities, additional analyses will be required to determine whether hyperlocomotion in *Elfn1* mutants indeed relates to dysfunction in habenular circuitry and to elucidate the nature of the underlying circuit defects.

*Elfn1* mutants also display reduced thigmotaxis, which is usually taken as a measure of anxiety levels and which is sensitive to dopaminergic signaling [87]. Though habenular function has been implicated in regulating anxiety levels [79], [84], [88], [89], this kind of phenotype could also be related to Elfn1 functions in any number of other regions, most obviously including the septum, extended amygdala and hippocampus [90], [91].

Collectively, our findings clearly demonstrate a requirement for Elfn1 *in vivo* for normal brain function. They suggest in addition that *Elfn1* mutants may model some aspects of the pathophysiology of ADHD. However, more behavioural analyses are required to test for impulsivity and attention deficits in order to fit the criteria for an optimal animal model of ADHD [58], [59], [60]. *Elfn1* mutants may also present an interesting model of epileptogenesis, with network disturbances arising over time. These may be attributable to defects in the specification of synaptic properties between specific pairs of cell-types in hippocampus or other brain regions [41], though the expression pattern and distribution of Elfn1 protein suggest the possibility of additional cellular functions. Whether removal of one copy of Elfn1 causes any more subtle or less penetrant phenotypes is an open question. We did observe several outliers amongst heterozygous *Elfn1* mutants in tests of locomotion and thigmotaxis (Figure 7C–F). Comparisons with *Elfn1^+/+^* littermates should reveal whether these represent normal variability or a heterozygous phenotype.

ADHD and epilepsy show higher than expected co-morbidity and overlapping genetic etiology [92], [93], [94], [95]; the prevalence of ADHD has been shown to be between 3–7% in children, whereas at least 20% of children with epilepsy also have ADHD [96]. It is interesting to note that developmental coordination disorder (DCD) also occurs in up to 50% of children with ADHD [97]. The spectrum presented in *Elfn1* mutant mice, which includes motor abnormalities, may thus be of clinical relevance. Neurodevelopmental disorders can arise due to mutations in any of a very large number of genes [98]. Given the phenotypes we observe in mice, it seems plausible that severe mutations in the human *ELFN1* gene (located at 7p22.3) could be involved in the etiology of rare cases of epilepsy, possibly involving symptoms of ADHD.

## Supporting Information

Figure S1 Elfn1 expression in the adult mouse brain. (A–H) Adult brain coronal sections from the Allen Brain Atlas show similar expression patterns as found at P0. (E) Close-up of area shown in white box in D. (G) Close-up of area shown in white box in F. AHP, anterior hypothalamic area (posterior part). (I–L) Adult brain sagittal sections from the Allen Brain Atlas show similar expression patterns as found at P0. Ctx, cortex; DBB, diagonal band of Broca; FR, fasciculus retroflexus; GP, globus pallidus; Hb, habenula; hc, hippocampus; Sb, subiculum; SN, substantia nigra; VP, ventral pallidum; hi, hilus of the dentate gyrus; lHb, lateral habenula; mHb, medial habenula; MS, medial septum; Pir, piriform cortex; Sb, subiculum; SNc, substantia nigra pars compacta; so, stratum oriens; sr, stratum radiatum; VP, ventral pallidum. Note that contrast was increased in these images using the Corrections function in Microsoft Powerpoint.
(TIF)

Figure S2 Elfn1 expression in the cortex at P0. (A,B) Double immunofluorescent staining on coronal sections at P0 with anti-Ctip2 and anti-β-gal shows that Elfn1 is not expressed in Ctip2-positive projection neurons. Ctip2 is highly expressed in layers 5 and 6, with strongest staining found in layer 5b. Elfn1-expressing cells are most intense in layers 4 and 5, but there is also a low number of scattered cells throughout the other layers (A). In the piriform cortex, Ctip2 expression is restricted to layer 2. Elfn1 is expressed in layer 3 and the endopiriform cortex (B).
(TIF)
