# Supplementary figures and images for: Mutation of Elfn1 in Mice Causes Seizures and Hyperactivity
Source: PLoS One. 2013 Nov 27;8(11):e80491. doi: 10.1371/journal.pone.0080491 (PMC3842350; doi:10.1371/journal.pone.0080491)

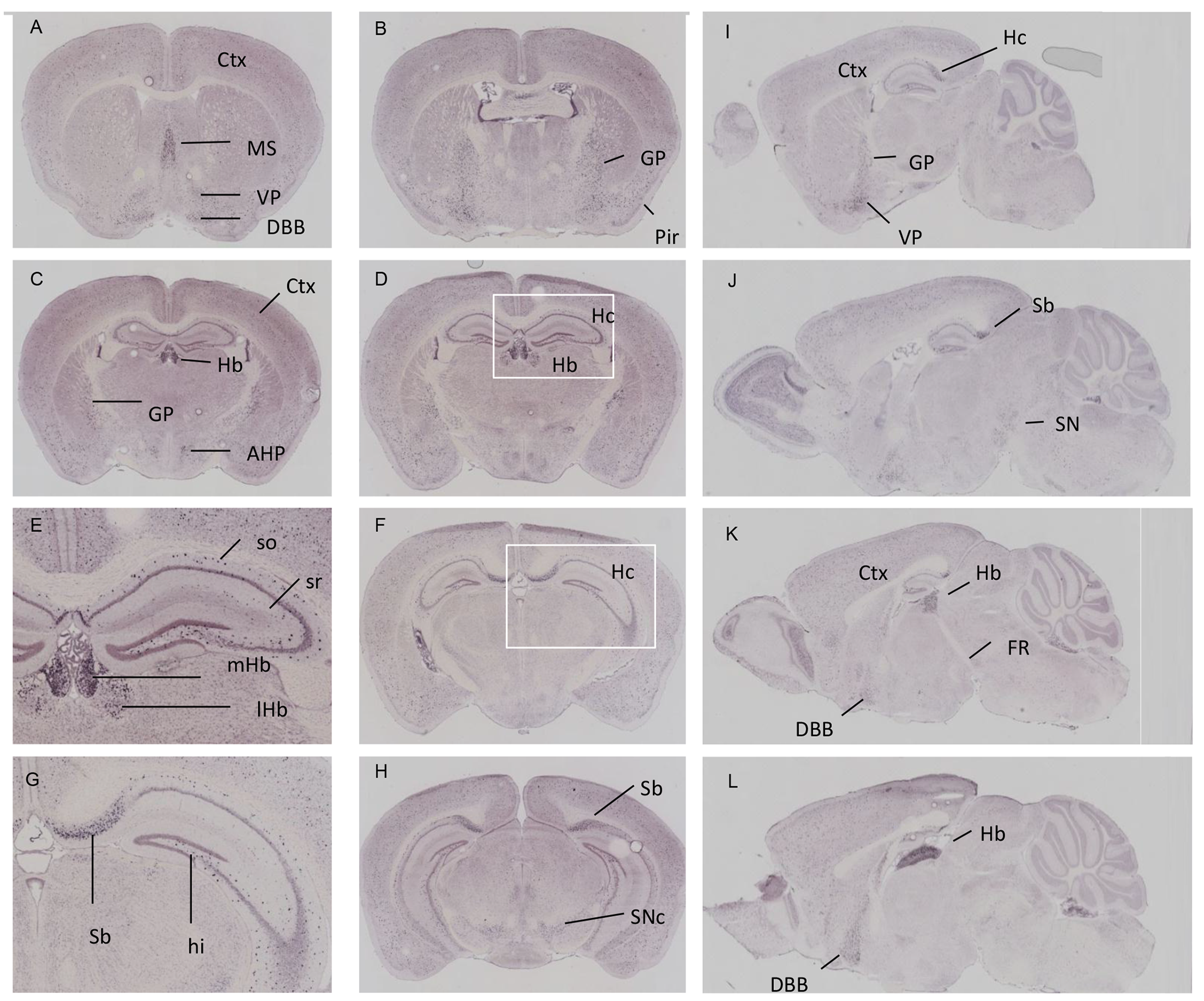

Supplement: Figure S1 — Elfn1 expression in the adult mouse brain. (A–H) Adult brain coronal sections from the Allen Brain Atlas show similar expression patterns as found at P0. (E) Close-up of area shown in white box in D. (G) Close-up of area shown in white box in F. AHP, anterior hypothalamic area (posterior part). (I–L) Adult brain sagittal sections from the Allen Brain Atlas show similar expression patterns as found at P0. Ctx, cortex; DBB, diagonal band of Broca; FR, fasciculus retroflexus; GP, globus pallidus; Hb, habenula; hc, hippocampus; Sb, subiculum; SN, substantia nigra; VP, ventral pallidum; hi, hilus of the dentate gyrus; lHb, lateral habenula; mHb, medial habenula; MS, medial septum; Pir, piriform cortex; Sb, subiculum; SNc, substantia nigra pars compacta; so, stratum oriens; sr, stratum radiatum; VP, ventral pallidum. Note that contrast was increased in these images using the Corrections function in Microsoft Powerpoint. (TIF) [file pone.0080491.s001.tif]

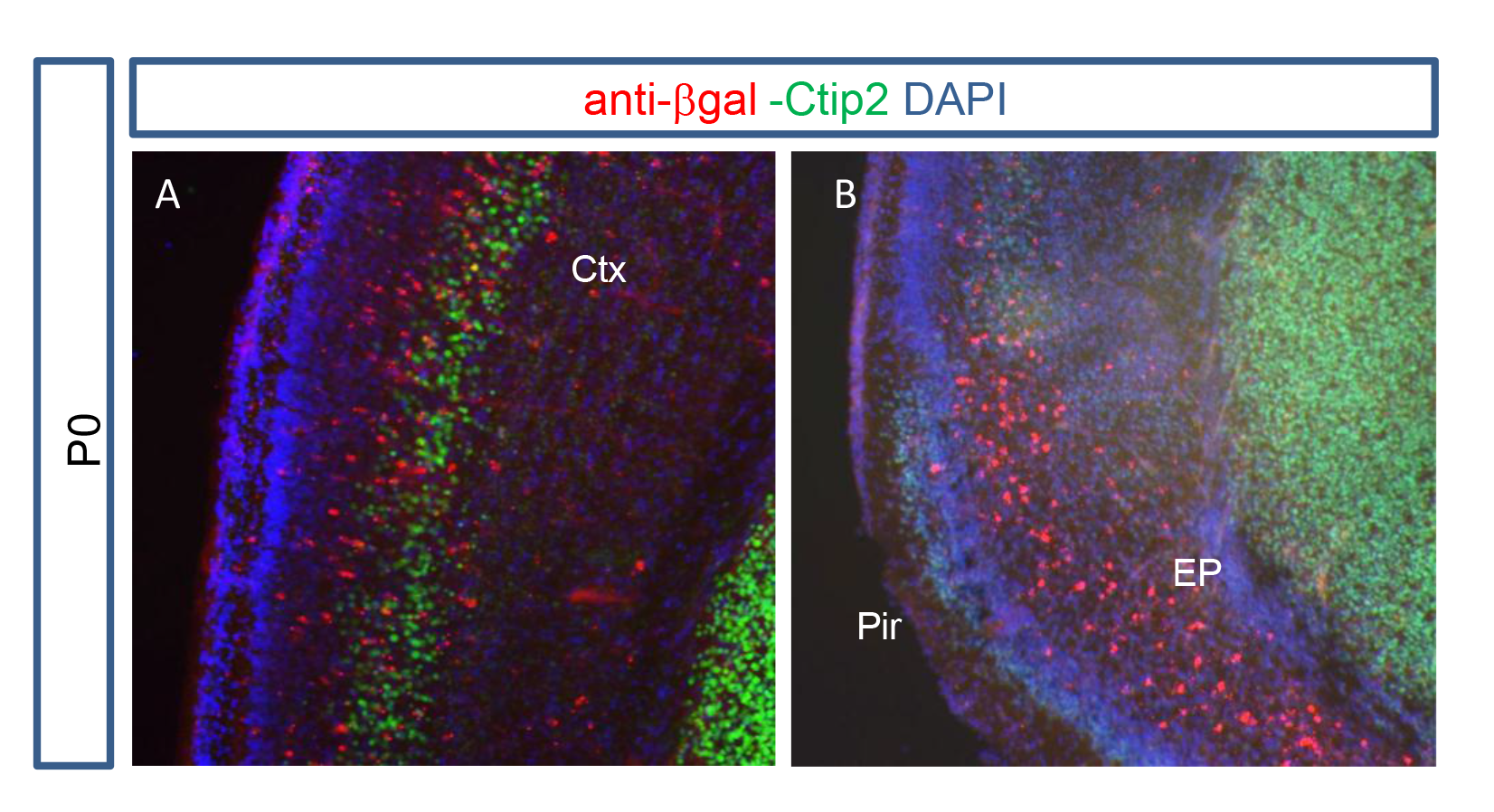

Supplement: Figure S2 — Elfn1 expression in the cortex at P0. (A,B) Double immunofluorescent staining on coronal sections at P0 with anti-Ctip2 and anti-β-gal shows that Elfn1 is not expressed in Ctip2-positive projection neurons. Ctip2 is highly expressed in layers 5 and 6, with strongest staining found in layer 5b. Elfn1-expressing cells are most intense in layers 4 and 5, but there is also a low number of scattered cells throughout the other layers (A). In the piriform cortex, Ctip2 expression is restricted to layer 2. Elfn1 is expressed in layer 3 and the endopiriform cortex (B). (TIF) [file pone.0080491.s002.tif]
